# The Use of Cyanoacrylate Tissue Adhesives in Various Wound Suturing Techniques to Enhance the Healing Process of Surgical Wounds: An Animal Study

**DOI:** 10.1002/cre2.70057

**Published:** 2025-03-07

**Authors:** Mojtaba Alijani, Shokofeh Jamshidi, Reza Nadripour, Naser Kamyari, Ali Heidari

**Affiliations:** ^1^ Faculty of Dentistry Hamadan University of Medical Sciences Hamadan Iran; ^2^ Department of Oral and Maxillofacial Pathology Hamadan University of Medical Sciences Hamadan Iran; ^3^ Department of Biostatistics and Epidemiology, School of Health Abadan University of Medical Sciences Abadan Iran; ^4^ Department of Oral and Maxillofacial Surgery Hamadan University of Medical Sciences Hamadan Iran

**Keywords:** cyanoacrylate, suture technique, tissue adhesive, wound healing

## Abstract

**Objectives:**

This study aimed to investigate the effect of cyanoacrylate tissue adhesive and suture techniques on closing surgical wounds in rabbits.

**Materials and Methods:**

This study randomly divided 32 male New Zealand white rabbits into four groups. In the first group, interrupted sutures were applied. In the second group, interrupted sutures were placed, and using a pipette, the surface of the wound was covered by tissue adhesive. In the third group, the wound was closed with a continuous suture, and in the fourth group, in addition to the continuous suture, tissue adhesive was also used. On the fifth day of surgery, rabbits were killed and tissue samples were collected and examined for epithelial tissue thickness, rate of inflammatory tissue, and area of granulation tissue.

**Results:**

In general, among the four groups, the highest thickness of the formed epithelial tissue was in the continuous suture without tissue adhesive group, and the lowest of epithelial tissue was seen in the interrupted suture with tissue adhesive group. There was a tendency to reduce the intensity of inflammation in the groups that used tissue adhesive, but except in one case, no significant difference was seen in the rest of the groups. Among the studied groups, the rate of granulation tissue was less in the continuous suture with an adhesive group compared with the other groups.

**Conclusions:**

The use of tissue adhesive on any type of suture technique can reduce the rate of inflammation and cause less granulation tissue. In the short term, the use of tissue adhesive could be an obstacle in the formation of epithelial tissue.

## 1 Introduction

Surgical treatments usually start with making a surgical incision and end with wound closure. The success of surgical treatment depends on the complete and uncomplicated closure of the surgical wounds. Various factors affect wound healing, which can be divided into two systemic and local groups (Guo and Dipietro 2010). If appropriate systemic conditions are provided, appropriate local conditions of the wound will help the success of the healing. A defining factor is the close proximity of the wound edges to each other and their impermeability to foreign substances (Ennis and Meneses 2000). The oral cavity and saliva have been considered to possess the most infectious areas of the body due to the presence of many microorganisms (Dawes et al. 2015; Pan, Zhao, and Jiang 2014). As long as the mucosal barrier of the oral cavity is healthy, these microorganisms may not result in contamination or infection, but as soon as they enter the underlying tissue layers through a wound, they can lead to inflammation and infection, causing problems in wound closure.

In intraoral surgeries, the impermeability of the wound to fluids in the oral cavity to the point of being watertight is emphasized. In particular, when placing allografts or even autografts, it is strongly recommended to put the edges of the wound together with high precision and create a completely impenetrable wound that prevents the infiltration of saliva (Hassan 2017). Different suture techniques have been used to achieve the goal of wound closure, each has advantages and disadvantages. Additionally, in today's commercial market, other materials have been introduced to help close the wound better and improve the healing process. One of these is cyanoacrylate tissue adhesives (CTA) with many advantages over traditional materials. This type of adhesive was approved by the Food and Drug Administration (FDA) in 1998 for the healing of surgical wounds caused by trauma (Hassan 2017). Some studies have reported that they can be used instead of the conventional suture techniques (Soni et al. 2013; Kumar et al. 2013). Some of the advantages of these adhesives include no tissue irritation, no tissue damage, ease of use, and antibacterial effects (Kumar et al. 2013). The successful application of these adhesives has been reported in orthopedic, neurological, gastrointestinal, and plastic surgeries (Bruns and Worthington 2000).

In recent years, extensive application of adhesives in the oral cavity has been considered, including closing wounds and oral fistulas or even fixing bone fragments (Borie et al. 2019). CTA are created with a combination of Cyanoacetate and formaldehyde; they are liquid monomers that, after exposure to a wet surface, undergo a heat‐generating reaction and are turned into a polymer that creates a strong tissue bond (Guhan et al. 2018; Ahn et al. 2011). These adhesives have high mechanical strength and can remain in place for several days (Simonova et al. 2012). Its most common types are 2‐octyl‐cyanoacrylate and n‐2‐butyl‐cyanoacrylate. 2‐Octyl cyanoacrylate is often preferred due to its higher flexibility (Ahn et al. 2011). Some studies have been conducted on the effects of these adhesives on skin incisions, but due to the different nature of mucosal wound healing compared with skin wounds, it has not been considered. Application of these adhesives with a special suture technique can effectively reduce the inflammation inside the wound. They can significantly increase the quality of wound closure and finally result in a more successful treatment. There are limited scientific sources that have simultaneously examined and compared the effects of suture techniques and the application of tissue adhesives on mucosal wounds. Thus, the present study was conducted to examine the effects of CTA in different suture techniques on the healing rate of the mucosal wound in the rabbit model.

## 2 Materials and Methods

The present study was approved by the research ethics committee of the university where the research was carried out and registered with the following code of ethics: IR.UMSHA.REC.1399.446. Since this research was carried out on laboratory animals, there was no need to obtain informed consent from the studied samples.

### 2.1 Sample Size Description

In this study, we used 32 male New Zealand white rabbits weighing between 2.5 and 3.5 kg.

For sample size calculation, the between‐subject error DF is considered in the formula (*n* = DF/*k* + 1), where *k* is the number of groups. Based on the acceptable range of the DF, the DF in the formulas is replaced with the minimum (10) and maximum (20) DFs to obtain the minimum and maximum numbers of animals per group (Wan Mohammad 2017). In conclusion, for the proposed study, between five and seven animals per group are required. In other words, a total of 20–28 animals are required to keep the DF within the range of 10–20. For more validity, we consider eight samples in each group.

Ten days before the surgery until the end of the study, animals were kept in a 12 h light/12 h dark (12:12 LD) cycle with food and water ad libitum. The place's temperature was 22 ± 3 and the air humidity was between 40% and 50%.

Rabbits were randomly divided into four groups. They were anesthetized using intraperitoneal Ketamine 10 mg/kg (Ketamine 10%, Alfasan, Woedren‐Holland) and Xylazine 3 mg/kg (Xylazine 2%, Alfasan, Woedren‐Holland). Immediately before the surgery, their oral cavity was disinfected with chlorhexidine (chlorhexidine‐Najo/2%, Tehran, Iran). Then, the tongues of the rabbits were numbed using 2% lidocaine + 1% epinephrine (persocaein‐E, Darupakhsh, Iran). A 20 mm surgical incision was made in the center of each rabbit's tongue using a measurement ruler and Surgical Blade No 15. The depth of incision includes mucosa and submucosa. In the first group, an interrupted suture was applied every 4 mm. In the second group, an interrupted suture was placed every 4 mm, and using a pipette, the surface of the wound was covered by a tissue adhesive, PeriAcryl 90HV (Glustitch Inc, Canada). In the third group, the wound was closed with a continuous suture, a needle passing every 4 mm. In the fourth group, in addition to the continuous suture, tissue adhesive was also used. Sutures were performed using chromic thread 04 (Supa, Iran) with a 16 mm reverse cutting needle. All surgeries were performed by an oral and maxillofacial surgeon with sufficient skill and under the same conditions. On the day of surgery and up to 2 days after it, cefazolin 20 mg/kg per day (AFA Chemic Pharma, Iran) was used as prophylaxis. To relieve pain, Ketorolac 5 mg/kg per day (Caspian Tamin Pharmaceutical, Tehran, Iran) was injected intraperitoneally. On the fifth day of surgery, rabbits were killed using an overdose of ketamine, and after completely washing the mouth with normal saline, a tissue sample was taken from the wound site. During the preparation of the tissue sample, two sutures were made on both sides of the central point of the scar tissue, and the examined slide was prepared from the center region of these two sutures. Then, the samples were immersed in 10% formalin. In the next step, a paraffin block was prepared from each sample, and then coronal sections were cut in three locations alongside the incision site, and slides were prepared so that the thickness of the mucosa, sub‐mucosa, and tongue muscles could be seen in one tissue section. The samples were stained with H&E and the histological examinations were evaluated and measured by an oral and maxillofacial pathology expert who was blinded to the coding and type of treatment in the samples. To control intra‐observer differences, 20% of slides were re‐evaluated by the same observer 2 weeks later and the data were the same as the initial evaluation in all cases.

The data obtained from the histological study were examined and statistically analyzed by three histopathological indicators including the rate of granulation tissue formation, the rate of inflammatory tissue, and the formation of epithelial tissue. Using Analysis LS Starter software (Olympus Soft Imaging Solutions), the area of the newly created granulation tissue was measured in square micrometers (µm²) with ×100 magnification and the thickness of the formed epithelium was measured in micrometers(µm) with ×40 magnification. To examine the rate of inflammatory cell infiltration in the studied groups, first, the area with the highest accumulation of inflammatory cells was identified using ×40 magnification, then the process was conducted with ×100 magnification. Then, they were graded based on the number of inflammatory cells in one field, each sample was considered to be in one of the following four categories: No inflammatory cells–mild inflammation (1–100 cells)–moderate inflammation (100–250 cells)–severe inflammation (more than 250 cells).

### 2.2 Statistical Methods

Descriptive statistics were analyzed using frequency tables, means, and standard deviations. The normality of the response distribution was tested using the Kolmogorov–Smirnov test. Due to the non‐normal distribution of responses, comparisons between treatment groups were performed nonparametrically using *χ* ^2^, Mann–Whitney, and Kruskal–Wallis tests. All statistical analyses were performed using SPSS v.21 software (IBM, Chicago, IL, USA) at a significance level of 0.05.

## 3 Results

### 3.1 Comparison in Terms of the Rate of Epithelial Tissue Formation

**Figure 1:**
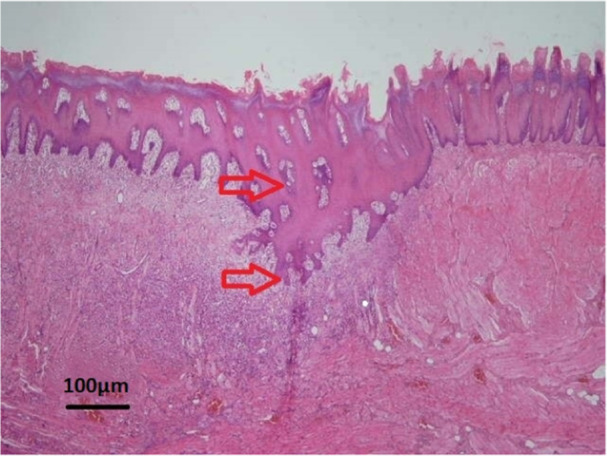
Microscopic view of the epithelium tissue formed with high thickness with H&E staining at ×40 magnification. The site of the surgical incision is marked with red arrows.

**Figure 2:**
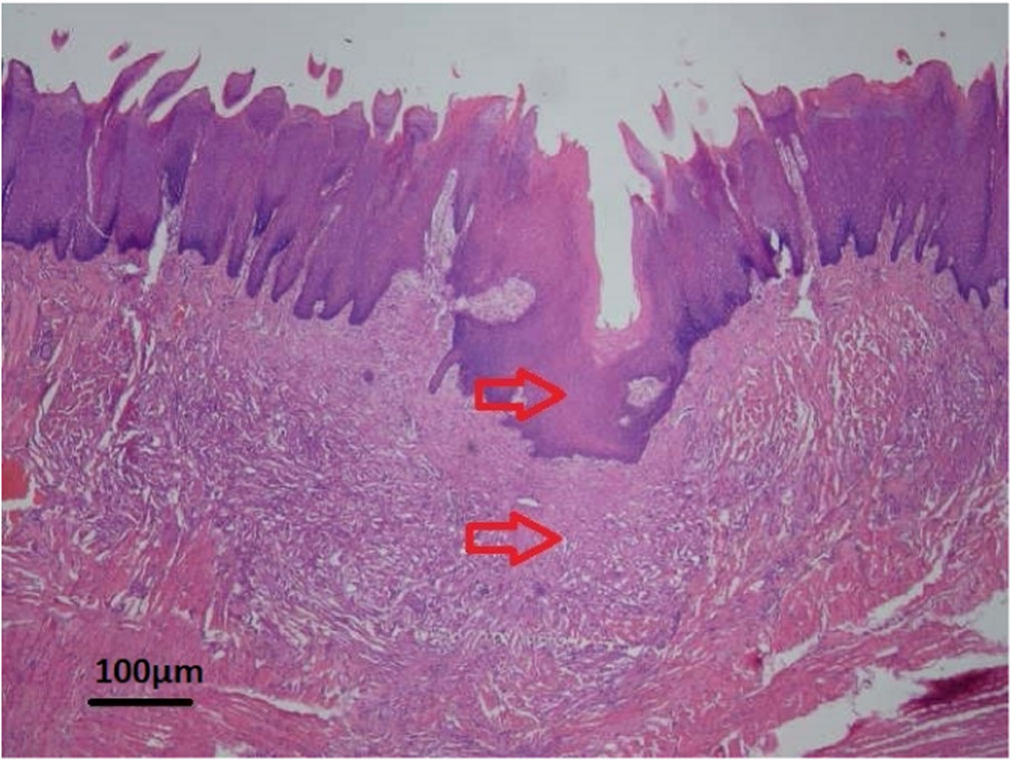
Microscopic view of the epithelium tissue formed with low thickness with H&E staining at ×40 magnification. The site of the surgical incision is marked with red arrows.

In general, among the four groups, the highest thickness of the formed epithelial tissue (Figure 1) was observed in the continuous suture without tissue adhesive group (368.58 ± 74.79) and the lowest of epithelial tissue (Figure 2) was seen in the interrupted suture with tissue adhesive group (185.42 ± 196.09). The second rank in terms of epithelial tissue formation belonged to the interrupted suture without adhesive group (362.9 ± 185.79) and the third rank belonged to the continuous suture with adhesive group (242.65 ± 135.25). However, in general, no statistically significant difference was found among the four groups in terms of the rate of epithelial tissue formation (*p* = 0.103). However, it seemed that the use of tissue adhesive reduced the epithelial thickness in wounds (Table 1).

**Table 1:** The results of measuring histological indicators.

		Group				
Variable		I (*N* = 8)	II (*N* = 8)	III (*N* = 8)	IV (*N* = 8)	*p*‐value
Epithelial thickness, mean ± SD(µm)		362.9 ± 185.79	185.42 ± 196.09	368.58 ± 74.79	242.65 ± 135.25	0.103^a^
Inflammatory infiltration, *N* (%)	Mild	0 (0.0%)	1 (8.3%)	3 (50.0%)	10 (83.3%)	< 0.001^b^
	Middle	2 (33.3%)	7 (58.3%)	3 (50.0%)	2 (16.7%)	
	Severe	4 (66.7%)	4 (33.3%)	0 (0.0%)	0 (0.0%)	
Granulation tissue area, mean ± SD (µm²)		656485.83 ± 238126.04	541170.09 ± 242615.32	523757.0 ± 95989.20	275073.91 ± 304647.66	0.050^a^

Abbreviations: I, simple non‐stick; II, simple with‐stick; III, continuous non‐stick; IV, continuous with‐stick; SD, standard deviation.

a *p‐*value carried out from Kruskal–Wallis test.

^b^ *p‐*value carried out from *χ* ^2^ test.

The pairwise comparison of the groups also revealed more understandable results (Table 2). There was no significant difference between the interrupted suture group (362.9 ± 185.79) and the interrupted suture with adhesive group (185.42 ± 196.09) in terms of epithelial tissue formation (*p* = 0.132), although the use of adhesive decreased the rate of epithelial tissue formation. There was a statistically significant difference between the continuous suture group (368.58 ± 74.79) and the continuous suture with adhesive group (242.65 ± 135.25) (*p* = 0.031); using adhesive materials could reduce the rate of epithelial tissue formation.

**Table 2:** Results (*p*‐value) for pairwise comparisons.

Variable	Paired groups			
	I vsII	III vs. IV	I vs. III	II vs. IV
Epithelial thickness	0.132	0.031	0.749	0.418
Inflammatory infiltration	0.326	0.343	0.070	0.003
Granulation tissue area	0.445	0.080	0.611	0.061

*Note: p*‐value carried out from the Mann–Whitney *U* test. Significance values have been adjusted by the Bonferroni correction for multiple tests.

Abbreviations: I, simple non‐stick; II, simple with‐stick; III, continuous non‐stick; IV, continuous with‐stick.

There was no significant difference (*p* = 0.749) in the interrupted suture without adhesive group (362.9 ± 185.79) and the continuous suture without adhesive group (368.58 ± 74.79), showing that the type of sutures did not make a significant difference in the rate of epithelial tissue thickness. After using adhesives, there was no significant difference between the interrupted suture with adhesive (185.42 ± 196.09) and the continuous suture with adhesive groups (242.65 ± 135.25) (*p* = 0.418). These results showed that although the suture type alone was not effective in the rate of epithelial tissue, after using tissue adhesive, the rate of epithelial tissue formation in the continuous suture with the adhesive group was higher than that of the interrupted suture with the adhesive group.

### 3.2 Comparison of Groups in Terms of Rate of Inflammatory Tissue Formation

A comparison of the four studied groups revealed that the rate of inflammatory tissue formation was as follows.

In the comparison of the groups in terms of the “use of tissue adhesive,” except in one case, no significant difference was seen in the rest of the groups, although there was a tendency to reduce the intensity of inflammation in the groups that used tissue adhesive.

Only in the comparison between the continuous suture group with tissue adhesive and the interrupted suture group with tissue adhesive, in the first group, the intensity of inflammation was significantly lower.

**Figure 3:**
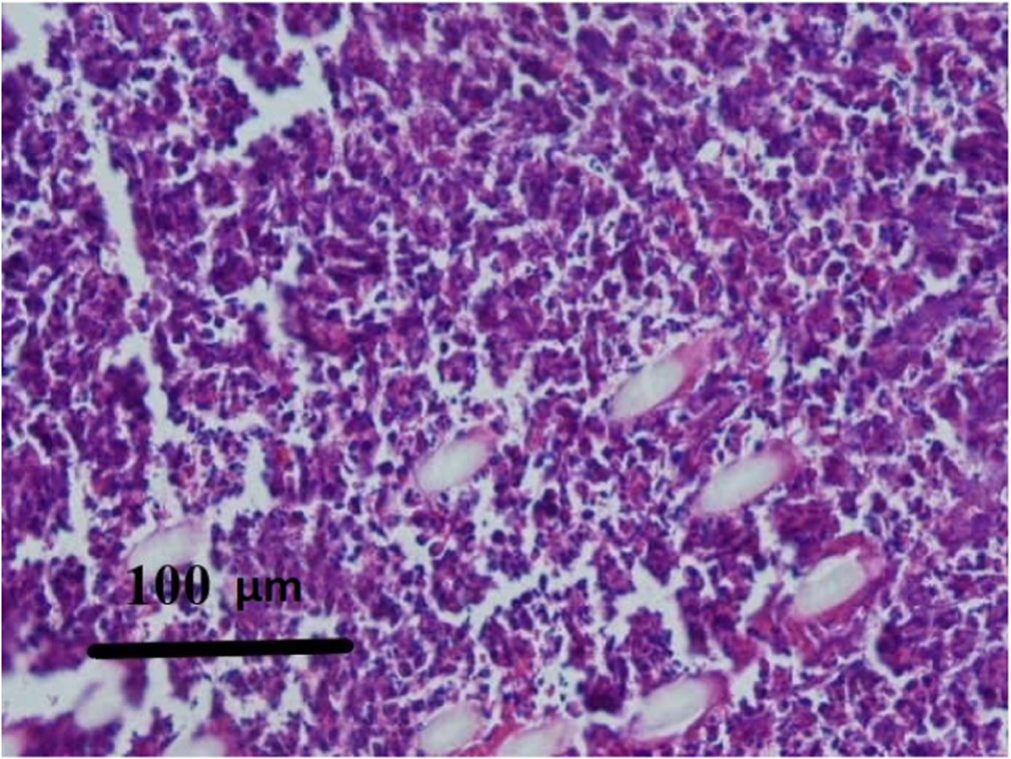
Microscopic view of inflammatory tissue 5 days after surgery. Severe infiltration and a high density of plasma cells and lymphocytes can be seen in the field. (H&E staining at ×100 magnification).

**Figure 4:**
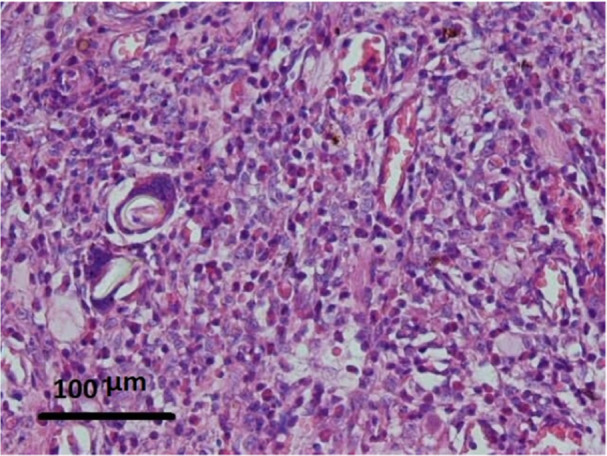
Microscopic view of inflammatory tissue 5 days after surgery. Moderate infiltration and density of plasma cells and lymphocytes can be seen in the field. (H&E staining at ×100 magnification).

In the interrupted suture group, 66.7% of samples had severe inflammation (Figure 3) and 33.3% of samples had moderate inflammation, and none showed mild inflammation. In the interrupted with tissue adhesive group, 33.3% of the samples showed severe inflammation, 58.3% showed moderate inflammation, and 8.3% showed mild inflammation (Figure 4).

In the continuous suture group, none of the samples showed severe inflammation, and the rate of moderate and mild inflammation was 50%. In the continuous suture with tissue adhesive group, none of the samples showed severe tissue inflammation, and the rate of moderate and mild inflammation were 16.7% and 83.3%, respectively (Table 1).

After pairwise comparisons in the rate of inflammatory tissue, it was found that although the use of tissue adhesive reduced the rate of inflammation to some extent, there was no significant difference comparing the interrupted suture group and the interrupted suture with an adhesive group (Table 2) (*p* = 0.326).

There was no significant difference in comparison between the continuous suture group and the continuous suture with an adhesive group (*p* = 0.343), although the use of adhesive caused a shift toward mild inflammation. The results of this study showed that although the use of tissue adhesive does not cause a significant difference in terms of the rate of wound inflammation, it has reduced its rate.

In comparing “all 4 groups together,” from the point of view of the “suturing technique,” it seems that in continuous suturing, the amount of inflammation is significantly lower than in interrupted suturing (*p* ≤ 0.001). But comparing the two groups together, the results were as follows.

There was no significant difference in the comparison between the interrupted suture and continuous suture groups (*p* = 0.070), although the use of continuous sutures reduced the inflammation distinctly.

In the comparison between the interrupted with the adhesive group and the continuous suture with the adhesive group, a significant difference was seen in terms of inflammation (*p* = 0.003), which showed that in terms of inflammation, the use of adhesive on sutures may cause a difference between the two suture techniques evidently.

The combination of continuous suture and tissue adhesive showed the highest reduction in tissue inflammation.

### 3.3 Comparison of Groups in Terms of the Rate of Granulation Tissue Formation

Among the four studied groups, the rate of granulation tissue was less in the continuous suture with an adhesive group (*p* = 0.050) compared with the other three groups (304,647 ± 275,073) and the rate of granulation tissue in the other three groups is as follows.

Continuous suture without an adhesive group (523,757 ± 95,989), interrupted suture with an adhesive group (541,170 ± 242,625), and interrupted suture without an adhesive group (656,485 ± 238,126) (Table 1).

These data showed that the simultaneous use of continuous suture and tissue adhesive has the greatest effect in reducing the rate of granulation tissue in the wound. The pairwise comparison of the groups in terms of the rate of granulation tissue formation also showed the following results.

In the comparison between the simple group and the simple with the adhesive group, no significant difference was seen (*p* = 0.445), although the use of adhesive caused some reduction in the granulation tissue.

In the comparison between the continuous suture group and the continuous with the adhesive group, no significant difference was observed (*p* = 0.080), although the difference between these two groups was more apparent than in the previous case and the rate of granulation tissue decreased in the presence of adhesive.

Although not statistically significant, comparing the two cases showed that the use of tissue adhesive can reduce the rate of granulation of tissue.

The comparison of interrupted suture and continuous suture groups did not show any significant difference (*p* = 0.611), although the rate of granulation tissue was less in the continuous suture.

Also, a comparison of the interrupted suture with an adhesive group with the continuous suture with an adhesive group showed that in the continuous tissue with the adhesive group, the rate of granulation tissue is obviously different from that of the interrupted suture with the adhesive group; however, these results were not statistically significant (*p* = 0.061) (Table 2).

## 4 Discussion

Wound closure is a major factor in improving tissue healing after surgery. The aim of wound repair or closing an incision is to close the edges of the wound, reduce the dead space, and minimize the risk of infection and contraction so that the healing process of the wound serves its functional and cosmetic goals (Suthar et al. 2020; Azmat and Council 2022). At present, the suture is the most common method used for intraoral wound closure. The most commonly used suture is the simple interrupted suture. Differences in wound closure methods can affect tissue healing outcomes. More trials with a lower risk of bias are needed to determine which types of sutures are better (Singh et al. 2019; Tacconi, Spinelli, and Signorelli 2019).

The drawback of the suturing may include extensive tissue damage which may cause bacterial access to the underlying tissues increasing the risk of infection. Moreover, suturing can cause complications such as suture loosening, abscess, epithelial cysts, wound leakage, permanent suture scars, foreign body reactions, tissue ischemia, fistula, and granuloma formation. In search of solving these problems, new methods and materials have been suggested. New biomaterials have been discovered as alternatives to conventional sutures over the years (Suthar et al. 2020; Azmat and Council 2022; Gurusamy et al. 2014; Gassner 2002; Liu et al. 2019; Deng et al. 2019; Singh et al. 2019). Tissue adhesives are an example of these materials, which are very useful since they are painless and act quickly. These adhesives can be a useful supplement for deeper stitches. They cause minimum wound inflammation and have a lower infection rate than sutures (Liu et al. 2019; Deng et al. 2019; Singh et al. 2019; Tacconi, Spinelli, and Signorelli 2019).

Cyanoacrylates have been among the most common tissue adhesives used in recent decades. They are biocompatible and have been studied in the past few years as an alternative to conventional sutures in wound closure (Ramalingam 2020). Although tissue adhesives have many advantages, their disadvantages, albeit limited, have been reported. Thus, further studies with different designs are necessary to make accurate conclusions on its advantages and disadvantages (Ramalingam 2020).

The present study was carried out on 32 rabbits. In the first group, after creating a mucosal incision, the resulting wound was closed with an interrupted suture. In the second group, in addition to closing the wound with an interrupted suture, tissue adhesive was used on the wound. In the third group, the continuous suture was used to close the wound and in the fourth group, in addition to continuous suture, tissue adhesive was also used to cover the wound. At the end of the fifth day, tissue samples were collected. We used histological examination, including the thickness of newly formed epithelial tissue, the rate of inflammatory tissue, and the granulation tissue area.

The present study revealed that the use of tissue adhesive can reduce the rate of inflammatory tissue and granulation, although this difference was not statistically significant in some cases. This difference can be due to the sealing effect of tissue glue and preventing the entry of oral fluids and bacteria in the oral cavity into the wound. In addition, the anti‐inflammatory effects of tissue adhesive can also be effective in reducing wound inflammation. Furthermore, the use of continuous sutures compared with interrupted sutures reduced the rate of inflammation and granulation tissue, although the difference was not significant. It may be due to the irritating effect of surgical thread knots in causing inflammation in the tissue. Moreover, the infiltration of inflammatory agents through oral secretions and through the space between the knots in interrupted sutures can be another reason for the creation of inflammatory tissues and more granulation in this group. The results show that the rate of inflammation is less in the presence of tissue adhesive. It seems that the simultaneous application of continuous sutures with fewer knots and tissue adhesive leads to a significant reduction of inflammation and the formation of granulation tissue in the wound.

Although the present study did not show a statistically significant difference among the studied groups in terms of the rate of epithelial tissue formation, it seems that the application of tissue adhesive on wounds prevented the formation of epithelial tissue. Since the samples were collected on the fifth day, it seems that the presence of adhesive might have delayed the regeneration of the epithelial tissue. This difference is especially obvious in the presence of continuous sutures and has led to a statistically significant difference. However, no significant difference was observed between the interrupted suture and continuous suture in terms of epithelial tissue formation, and it seems that the type of suture technique does not play a significant role in this regard.

In a study conducted by Üstün et al. (2020), a single linear incision was made on the back of the tongue of 24 rats. It was shown that on Day 5 of this study, there was no difference in the results between suture and cyanoacrylate adhesive in terms of epithelial regeneration, as in our study. The rate of inflammation was higher in the tissue adhesive group, which contradicts the results of our study which could be due to the type of thread used. In the current study, vicryl thread was used, which might be the cause of this difference due to its nature (Üstün et al. 2020).

Our study is similar to the study conducted by Ahn et al. (2011), in which wounds were created on the eyelids of eight rabbits and showed that the inflammation caused by the application of CTA was less than the inflammation caused by sutures. In this study, no other significant histopathological difference was observed in the first week (Ahn et al. 2011). In a study conducted by Feritas et al. the results of using cyanoacrylate adhesive were compared with nylon thread; histopathological data showed that there was no significant difference between the two groups. In this study, the rate of infection was the same in two groups. However, in our study, none of the animal samples had an infection at the wound site (Freitas‐Júnior et al. 2008).

In a study on 15 endodontic patients, Giray et al. (1997) showed that the inflammation rate was higher on the side with sutures compared to the side with cyanoacrylate adhesive 7 days after surgery (Giray et al. 1997). The results mentioned were similar to those of our study in terms of the presence of inflammation, but it was different from our study in terms of the degree of epithelialization. However, confirmation in our study was taken from histopathological examination, but the base of mentioned study was clinical observations. Moreover, the above study assigned more time compared to our study (5 days) to regenerate the epithelium.

Similar to our study, in a study on intraoral wounds of four dogs, Eriksson (1976) showed that the use of tissue adhesive led to a delay in the process of epithelialization of the wound compared to sutures (Eriksson 1976). Kumar et al. (2013) in a study on 10 endodontic patients showed that the side treated with sutures had more inflammatory infiltration than the side treated with cyanoacrylate 7 days after surgery (Kumar et al. 2013), which is evidently similar to the results of the present study.

In a study on 30 alveoplasty patients, Vastani and Maria (2013) showed that the rate of inflammatory tissue infiltration and vascularization 7 days after surgery was higher in the suture group than in the group treated with cyanoacrylate adhesive, but it was not true 14 days after the surgery and only the vascularization of the suture group was still higher. Accordingly, they concluded that CTA is more effective in primary healing. Also, they did not find any difference in terms of fibroblastic activity on the two surgical sides (Vastani and Maria 2013). In our study, it seems that the duration of 5 days has caused the difference in epithelialization between the two groups with and without adhesive, and if more time is given, the results may change.

In a study on 24 periodontal pocket patients requiring flap surgery, Kulkarni, Dodwad, and Chava (2007) showed that the rate of inflammatory cells infiltration was high both in the suture site and in the cyanoacrylate adhesive site 7 days after surgery, but this rate of infiltration, as in our study, was more on the suture side than on the adhesive side (Kulkarni, Dodwad, and Chava 2007).

## 5 Conclusion

The use of tissue adhesive on any type of suture technique can reduce the rate of inflammation and cause less granulation tissue, which is considered a significant advantage in healing wounds. Although in the short term, the use of tissue adhesive could be an obstacle in the formation of epithelial tissue, this effect may not be continued in the long run. In clinical conditions, the use of tissue adhesives alone or as an auxiliary tool can be effective in improving the quality of wound healing by reducing tissue inflammation. In cases where the speed of epithelialization is a priority, they should be used with caution.

The suture technique does not have a significant role in the tissue indicators of the wound.

### 5.1 Limitations and Suggestions

Evaluating the wound healing process in a longer period of time and investigating the use of tissue adhesive alone and in a separate group can strengthen similar studies. Performing mechanical tests such as tensile strength could increase the power of the study. However, because performing these tests would change the integrity of the tissue sample and make it impossible to check the histological indicators carefully, it was omitted.

## Author Contributions

Mojtaba Alijani, Shokofeh Jamshidi, and Reza Nadripour performed the experiments. Ali Heidari and Shokofeh Jamshidi supervised the study. All authors read and approved the final manuscript.

## Disclosure

The lead author AH affirms that this manuscript is an honest, accurate, and transparent account of the study being reported; that no important aspects of the study have been omitted; and that any discrepancies from the study as planned (and, if relevant, registered) have been explained.

## Ethics Statement

The study was approved by the research ethics committee of the Hamadan University of Medical Sciences (IR. UMSHA. REC.1399.446).

## Conflicts of Interest

The authors declare no conflicts of interest.

## Data Availability

Data supporting this study are included within the article.

## References

[cre270057-bib-0001] Ahn, H. B. , D. M. Shin , M. S. Roh , W. J. Jeung , W. C. Park , and S. H. Rho . 2011. “A Comparison of 2‐Octyl Cyanoacrylate Adhesives Versus Conventional Suture Materials for Eyelid Wound Closure in Rabbits.” Korean Journal of Ophthalmology 25, no. 2: 121–127. 21461225 10.3341/kjo.2011.25.2.121 PMC3060389

[cre270057-bib-0002] Azmat, C. E. , and M. Council . 2022. “Wound Closure Techniques.” In StatPearls. Treasure Island (FL): StatPearls Publishing. 29262163

[cre270057-bib-0003] Borie, E. , E. Rosas , G. Kuramochi , S. Etcheberry , S. Olate , and B. Weber . 2019. “Oral Applications of Cyanoacrylate Adhesives: A Literature Review.” BioMed Research International 2019: 1–6. 10.1155/2019/8217602 PMC6441539 31008113

[cre270057-bib-0004] Bruns, T. B. , and J. M. Worthington . 2000. “Using Tissue Adhesive for Wound Repair: A Practical Guide to Dermabond.” American Family Physician 61, no. 5: 1383–1388. 10735344

[cre270057-bib-0005] Dawes, C. , A. M. L. Pedersen , A. Villa , et al. 2015. “The Functions of Human Saliva: A Review Sponsored by the World Workshop on Oral Medicine VI.” Archives of Oral Biology 60, no. 6: 863–874. 25841068 10.1016/j.archoralbio.2015.03.004

[cre270057-bib-0006] Deng, J. , Y. Tang , Q. Zhang , et al. 2019. “A Bioinspired Medical Adhesive Derived from Skin Secretion of *Andrias Davidianus* for Wound Healing.” Advanced Functional Materials 29, no. 31: 482–494.

[cre270057-bib-0007] Ennis, W. J. , and P. Meneses . 2000. “Wound Healing at the Local Level: The Stunned Wound.” Ostomy/Wound Management 46, no. 1A Suppl: 39. 10732639

[cre270057-bib-0008] Eriksson, L. 1976. “Cyanoacrylate for Closure of Wounds in the Oral Mucosa in Dogs.” Odontologisk Revy 27, no. 1: 19–24. 787859

[cre270057-bib-0009] Freitas‐Júnior, R. , R. R. Paulinelli , R. M. Rahal , et al. 2008. “The Use of 2‐octyl Cyanoacrylate or Nylon Suture for Skin Closure Produces Similar Modifications in Scar Tissue (An Animal Model).” Journal of Plastic, Reconstructive & Aesthetic Surgery: JPRAS 61, no. 8: 990–992. 10.1016/j.bjps.2007.11.052 18472319

[cre270057-bib-0010] Gassner, R. 2002. “Wound Closure Materials.” Oral and Maxillofacial Surgery Clinics of North America 14, no. 1: 95–104. 18088613 10.1016/s1042-3699(02)00009-2

[cre270057-bib-0011] Giray, C. B. , A. Atasever , B. Durgun , and K. Araz . 1997. “Clinical and Electron Microscope Comparison of Silk Sutures and n‐Butyl‐2‐Cyanoacrylate in Human Musosa.” Australian Dental Journal 42, no. 4: 255–258. 9316313 10.1111/j.1834-7819.1997.tb00130.x

[cre270057-bib-0012] Guhan, S. , S. L. Peng , H. Janbatian , et al. 2018. “Surgical Adhesives in Ophthalmology: History and Current Trends.” British Journal of Ophthalmology 102, no. 10: 1328–1335. 29581352 10.1136/bjophthalmol-2017-311643

[cre270057-bib-0013] Guo, S. , and L. A. Dipietro . 2010. “Factors Affecting Wound Healing.” Journal of Dental Research 89, no. 3: 219–229. 20139336 10.1177/0022034509359125 PMC2903966

[cre270057-bib-0014] Gurusamy, K. S. , C. D. Toon , V. B. Allen , and B. R. Davidson . 2014. “Continuous Versus Interrupted Skin Sutures for Non‐Obstetric Surgery.” Cochrane Database of Systematic Reviews, no. 2: CD010365. 24526375 10.1002/14651858.CD010365.pub2 PMC10692401

[cre270057-bib-0015] Hassan, H. 2017. “Koshak. Dental Suturing Materials and Techniques.” Glob J Otolaryngology 12, no. 2: 28–37.

[cre270057-bib-0016] Kulkarni, S. , V. Dodwad , and V. Chava . 2007. “Healing of Periodontal Flaps When Closed With Silk Sutures and N‐Butyl Cyanoacrylate: A Clinical and Histological Study.” Indian Journal of Dental Research 18, no. 2: 72–77. 17502712 10.4103/0970-9290.32424

[cre270057-bib-0017] Kumar, M. S. , S. Natta , G. Shankar , S. H. Reddy , D. Visalakshi , and G. V. Seshiah . 2013. “Comparison Between Silk Sutures and Cyanoacrylate Adhesive in Human Mucosa—A Clinical and Histological Study.” Journal of International Oral Health: JIOH 5, no. 5: 95–100. 24324311 PMC3845291

[cre270057-bib-0018] Liu, Y. , S. Cheong Ng , J. Yu , and W. B. Tsai . 2019. “Modification and Crosslinking of Gelatin‐Based Biomaterials as Tissue Adhesives.” Colloids and Surfaces B: Biointerfaces 174: 316–323. 30472617 10.1016/j.colsurfb.2018.10.077

[cre270057-bib-0019] Pan, J. , J. Zhao , and N. Jiang . 2014. “Oral Cavity Infection: An Adverse Effect After the Treatment of Oral Cancer in Aged Individuals.” Journal of Applied Oral Science 22, no. 4: 261–267. 25141196 10.1590/1678-775720130546 PMC4126820

[cre270057-bib-0020] Ramalingam, D. 2020. “Cyanoacrylae Adhesive in Oral Surgery—A Review.” European Journal of Molecular & Clinical Medicine. 7, no. 3: 1890–1893.

[cre270057-bib-0021] Simonova, G. , C. M. Rickard , K. R. Dunster , D. J. Smyth , D. McMillan , and J. F. Fraser . 2012. “Cyanoacrylate Tissue Adhesives—Effective Securement Technique for Intravascular Catheters: In Vitro Testing of Safety and Feasibility.” Anaesthesia and Intensive Care 40, no. 3: 460–466. 22577911 10.1177/0310057X1204000311

[cre270057-bib-0022] Singh, P. K. , S. Degala , S. Shetty , V. S. Rai , and A. Das . 2019. “To Evaluate the Efficacy and Effectiveness of N‐butyl‐2‐cyanoacrylate Glue (TRU SEAL) in Closure of Oral and Maxillofacial Laceration and Surgical Incisions.” Journal of Maxillofacial and Oral Surgery 18, no. 1: 131–138. 30728704 10.1007/s12663-018-1111-6 PMC6328818

[cre270057-bib-0023] Soni, A. , R. Narula , A. Kumar , M. Parmar , M. Sahore , and M. Chandel . 2013. “Comparing Cyanoacrylate Tissue Adhesive and Conventional Subcuticular Skin Sutures for Maxillofacial Incisions‐‐A Prospective Randomized Trial Considering Closure Time, Wound Morbidity, and Cosmetic Outcome.” Journal of Oral and Maxillofacial Surgery 71, no. 12: 2152.e1–2152.e8. 10.1016/j.joms.2013.08.029 24237777

[cre270057-bib-0024] Suthar, P. , S. Shah , P. Waknis , G. Limaye , A. Saha , and P. Sathe . 2020. “Comparing Intra‐Oral Wound Healing After Alveoloplasty Using Silk Sutures and N‐Butyl‐2‐Cyanoacrylate.” Journal of the Korean Association of Oral and Maxillofacial Surgeons 46, no. 1: 28–35. 32158678 10.5125/jkaoms.2020.46.1.28 PMC7049767

[cre270057-bib-0025] Tacconi, L. , R. Spinelli , and F. Signorelli . 2019. “Skin Glue for Wounds Closure in Brain Surgery: Our Updated Experience.” World Neurosurgery 121: e940–e946. 30336296 10.1016/j.wneu.2018.10.023

[cre270057-bib-0026] Üstün, O. , T. L. Kumral , Y. Atar , et al. 2020. “Histopathological Comparison of 2‐Octyl Cyanoacrylate and Primary Suturing for Tongue Lacerations.” Journal of Craniofacial Surgery 31, no. 4: e334–e337. 32176002 10.1097/SCS.0000000000006254

[cre270057-bib-0027] Vastani, A. , and A. Maria . 2013. “Healing of Intraoral Wounds Closed Using Silk Sutures and Isoamyl 2‐Cyanoacrylate Glue: A Comparative Clinical and Histologic Study.” Journal of Oral and Maxillofacial Surgery 71, no. 2: 241–248. 23089654 10.1016/j.joms.2012.08.032

[cre270057-bib-0028] Wan Mohammad, W. M. Z. 2017. “Sample Size Calculation in Animal Studies Using Resource Equation Approach.” Malaysian Journal of Medical Sciences 24, no. 5: 101–105. 10.21315/mjms2017.24.5.11. PMC5772820 29386977

